# Patient relevant endpoints in oncology: current issues in the context of early benefit assessment in Germany

**DOI:** 10.1186/2191-1991-4-2

**Published:** 2014-01-24

**Authors:** Inna Dabisch, Jürgen Dethling, Charalabos-Markos Dintsios, Melanie Drechsler, Daniel Kalanovic, Peter Kaskel, Frank Langer, Jörg Ruof, Thorsten Ruppert, Daniel Wirth

**Affiliations:** 1 German Association of Research-based Pharmaceutical Companies (vfa), Berlin, Germany; 2 GlaxoSmithKline GmbH & Co. KG, Munich, Germany; 3 Boehringer Ingelheim Pharma GmbH & Co. KG, Ingelheim, Germany; 4 Pfizer Pharma GmbH, Berlin, Germany; 5 MSD SHARP & DOHME GMBH, Lindenplatz 1, 85540, Haar, Germany; 6 Lilly Deutschland GmbH, Bad Homburg, Germany; 7 Roche Pharma AG, Grenzach-Wyhlen, Germany; 8 Janssen-Cilag GmbH, Neuss, Germany

**Keywords:** AMNOG, German health care reform, Oncology, Endpoints, Progression-free survival, Patient-relevant benefit

## Abstract

The German AMNOG healthcare reform includes a mandatory early-benefit-assessment (EBA) at launch. As per German social code, EBA is based on registration trials and includes evaluation of the patient-relevant effect of the new medicines compared to an appropriate comparator as defined by the Federal Joint Committee (G-BA). Current EBA decisions released have unveiled issues regarding the acceptance of some patient-relevant endpoints as G-BA and IQWiG are grading the endpoints, focusing on overall survival as the preferred endpoint in oncology.

A taskforce of experienced German outcomes research, medical, health-technology assessment and biostatistics researchers in industry was appointed. After agreement on core assumptions, a draft position was prepared. Input on iterative versions was solicited from a panel of reviewers from industry and external stakeholders.

Distinctive features of registration trials in oncology need to be considered when these studies form basis for EBA, especially in cancer-indications with long post-progression survival; and with several consecutive therapeutic options available post-progression. Ethical committees, caregivers and patients often demand cross-over-designs diluting the treatment-effect on overall survival. Regulatory authorities require evaluation of morbidity-related study endpoints including survival of patients without their disease getting worse (i.e., progression-free survival). Also, progression requires treatment-changes, another strong indicator for its relevance to patients.

Based on specific guidelines and clinical trial programs that were developed to be consistent with regulatory guidance, endpoints in oncology are thoroughly evaluated in terms of their patient-relevance. This extensive knowledge and experience should be fully acknowledged during EBA when assessing the patient-relevant benefit of innovative medicines in oncology.

**JEL codes:**

D61; H51; I18.

## Introduction

### Problem statement

Due to the severity of oncological diseases, pivotal studies in oncology are subject to special methodological characteristics that must be taken into account during the interpretation of the results. For ethical reasons, not every study design that is theoretically possible can be implemented in practice [1]. The European authorities (European Medical Agency, EMA), the higher federal authorities in Germany (Bundesinstitut für Arzneimittel und Medizinprodukte, BfArM, and Paul-Ehrlich-Institute, PEI, respectively) and ethics-committees in charge have defined specific requirements in this respect [2-4]. The benefit-assessment of a therapy in terms of reimbursement issues should also take into account these specific pre-requisites as part of the early-benefit-assessment (EBA), as conducted by German HTA (i.e., Institute for Quality and Efficiency in Health Care, IQWiG) as commissioned by the Federal Joint Committee (G-BA), or G-BA itself for orphan drugs). Assessments conducted based on the endpoint of overall survival (OS) alone would lead to results that put patients at a disadvantage [5]. This position paper demonstrates the basic positions of the authors in this regard, especially in terms of patient-relevant endpoints (PRE) in clinical trials.

### Task force process

A task force to further evaluate endpoints in oncological diseases, and survival endpoints in particular, under the auspices of the German Association of Research-based Pharmaceutical Companies (vfa) was appointed. Members were experienced German outcomes research, medical, health technology assessment (HTA) and biostatistics researchers in industry. In a first step, after definition of topics of interest, and text structure, respectively, author-teams were assigned (i) to further evaluate endpoints in oncology; (ii) to provide an integrated view on HTA, regulatory, medical and patients’ view; (iii) to further evaluate clinical value of survival without disease getting worse (i.e., progression-free survival, PFS); and (iv) to review HTA decisions on oncologic products in Germany. After structured literature research had been performed and draft versions on the pre-defined text topics had been created, manuscript was shortened and a final integrated structure of the manuscript was consented in a face-to-face meeting. Afterwards, a second draft was prepared, and input solicited from a panel of reviewers from industry and external stakeholders. Input obtained during a poster presentation at the European Meeting of the International Society for Pharmacoeconomics and Outcomes Research (ISPOR) in Berlin on Nov-6^th^, 2012 (PCN 152) was incorporated into the final version of the manuscript. In a last step, an approval of the final version was obtained from all authors.

## Review

### Marketing authorization (MA) of oncologic agents and clinical considerations

MA procedures for new pharmaceuticals in oncology review the therapeutic benefit:

The MA of a new pharmaceutical and/or the extension of the indication of an already approved drug are strictly regulated legally and at various levels. All national European regulatory agencies (including the BfArM and PEI) and the EMA demand proof of efficacy and safety resulting in a positive benefit-risk-ratio, and the clinical trial results must be submitted for each pharmaceutical. How clinical trials are conducted is stipulated in the German Medicinal Products Act, [3] and the higher EU directive 2001/20/EU, [6] respectively. Furthermore, a large number of international and European guidelines as part of the MA procedure must be heeded [2].

The situation of oncological patients–and also the aims of a clinical trial–is marked by great heterogeneity:

The categories for characterizing this heterogeneity in oncology are [7]:

• curative–palliative

• palliative: different disease stages

• palliative: symptomatic-asymptomatic

• palliative: very different median life expectancy of months (pancreatic cancer) to decades (indolent lymphoma)

• palliative: well treatable (breast cancer: more than 10 approved pharmaceuticals) vs. non-treatable

Clinical trials in oncology, which represent the basis for MA of pharmaceuticals, must take into account the fact that the patients typically suffer from life-threatening diseases. For ethical reasons and due to medical needs, a new therapy that has not yet been fully investigated in terms of efficacy and safety is often tested first on patients with advanced disease for whom the established therapies have failed [8]. Among the endpoints of the studies based on which efficacy is measured, OS is recognized as the most important endpoint [9]. At the same time, however, the determination of OS to evaluate the effect of a therapy often hits ethical limits and in many situations it is impossible or only possible in limited fashion for methodological reasons (e.g. due to different active follow-up therapies [10,11]). This is illustrated by several examples:

Oncological diseases with a long survival time: In part, the analysis of OS requires long follow-up monitoring periods, which delay the development of additional effective substances or make the implementation of clinical trials impossible, because in some cases the endpoint can only be reached after many years or decades. For example, chronic myeloid leukemia (CML) is a disease for which survival benefits can only be estimated through long-term evaluations after more than a decade [12]. The same may apply for a new pharmaceutical for metastasized breast cancer [13]. Due to the long survival time, an extension of OS could theoretically be proven through studies many years after launch. However, this will not be possible in practice, because it can be problematic to retain a sufficient number of patients in randomized clinical trials over longer periods of time, and long-term follow-up data are evaluated with caution among HTA researchers [14,15]. This particularly applies to oncological diseases for which multiple therapy-options are used sequentially. In these cases, survival is influenced by subsequent therapies, which makes it more difficult to judge the effect of the pharmaceutical substance used first on the OS endpoint. At a minimum it dilutes any effect [16]. Examples include metastasizing entities such as breast- and lung cancer [17,1].

### Survival-related and other endpoints in oncology: an integrated view

The selection of the primary endpoint in clinical trials in oncology and the cross-over question (Table 1) are typically the subject of intense discussion between researchers, study groups, regulatory bodies and ethics committees. The EMA has defined specific endpoints that are acceptable from a regulatory standpoint in its “Guideline on the evaluation of anticancer medicinal products in man” [2]. With regard to survival time, the regulatory agencies will demand OS as a primary endpoint if the substance to be tested is probably more toxic than the comparator, if no accepted therapies are available for further treatment and if the time from disease progression to death is short. If a different endpoint is chosen, this must be justified in detail to the regulatory agencies. In this context, survival of patients without their disease getting worse (i.e., PFS) is recognized as a clinically valid endpoint for survival and will represent an acceptable primary endpoint in the eyes of the regulatory agencies e.g. if subsequent therapies have an impact on OS [2]. Outcomes for PFS in cancer patients are available more quickly than for OS, and are not influenced by additional therapies after disease progression. Besides PFS, Disease-free survival (DFS) is regarded by the regulatory agencies as an appropriate endpoint to evaluate adjuvant therapies [2]. Other endpoints such as “complete remission” (CR) are recognized in the treatment of oncological diseases, the endpoint “objective response rate” (ORR) is used e.g. for brain tumors. Typically, in these situations OS must also be captured (as a secondary endpoint) with the objective that the new therapy does not create a disadvantage in this respect [2]. Considerations made by the regulatory agencies based on medical and scientific criteria and the endpoints of the pivotal clinical trials, which have been specified beforehand, must also be comprehensively taken into account and recognized during the EBA by G-BA or IQWiG (if commissioned by G-BA). Currently, this is not the case (Additional file 1: Table S1).

**Table 1:** Clinical trials for which ethics commissions, physicians and patients demand cross-over designs, i.e. the option of switching to the therapy of the other study arm

•	A considerable efficacy advantage compared to the standard therapy can be expected for the substance to be tested
•	A considerable efficacy advantage is demonstrated in an interim analysis e.g. with regard to a benefit in survival of patients without their disease getting worse (i.e., progression-free survival, PFS).
•	The decision to switch typically is being made at the individual patient level.
	◦ As soon as a benefit in terms of the efficacy of the tested substance compared to the comparator becomes apparent, patients wish to be treated with the new therapy.
	◦ For ethical reasons, this must therefore be made possible, even if and especially if the survival time analysis is influenced by it.
	◦ No trial participant can be asked to be treated with an inferior therapy until the end of his or her life, only to make the superiority of the better therapy more clearly apparent in terms of overall survival (OS).
•	Even if a trial does not provide for a cross-over, e.g. because the superiority of the new therapy is still uncertain, trial participants can terminate their trial participation at any time in order to be treated with the new medication (e.g. if it is already approved for another indication) or with similar pharmaceuticals.
	◦ Even this unplanned cross-over influences the survival time analysis (in a conservative direction).

Apart from OS, especially PFS must be mentioned with regard to mortality. If these endpoints – agreed upon with the regulatory agencies for assessing the benefit of a new therapy–are not considered accordingly, as part of the EBA, an assessment of the additional benefit can hardly be achieved in an appropriate manner.

**Figure 1:**
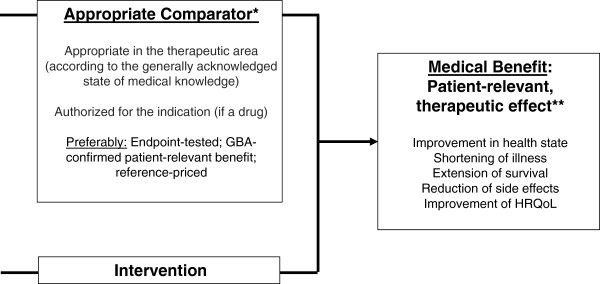
**Key AMNOG terms, as per German Social Code (Book V).** *or indirect comparison; **or *validated* surrogate. *AMNOG=Arzneimittelmarktneuordnungsgesetz (National health care law). HRQoL=Health related quality of life. G-BA=German Joint Federal Committee.*

An improvement in terms of morbidity is also considerably relevant for the patients:

Pursuant to the German Social Code, [18] insured patients are entitled to treatment, if it is necessary to diagnose a disease, cure it, prevent it from worsening or alleviate its symptoms. Oncological treatments often achieve an improvement in health state that is limited in time, but this improvement does not necessarily manifest itself in an extension of OS. Insured patients in Germany are also entitled to treatment, if it provides relief from their suffering. Currently, during EBA in Germany as performed by G-BA and IQWiG using HTA methodology, the treatment of tumor entities is predominantly held up to the standard criteria of a cure (without assigning any ratings) in terms of improved mortality, alleviation of symptoms (morbidity) and an improvement in the patients‘ health related quality of life (HRQoL; Figure 1). However, many diseases in oncology have no curative treatment in the foreseeable future. This includes e.g. advanced non-small cell lung cancer (NSCLC), renal cell carcinoma (RCC) and some malignant hematological diseases. Any HTA assessment conducted based on the endpoint of OS alone would lead to results that put the patients at a disadvantage within the context of EBA in Germany [5]. Although the levels of patient-relevant benefit (PRB) of EBA have been defined (Table 2), it must be emphasized that a definition of the term “patient relevance” and a final weighting of endpoints have yet to be made. Specifically, it must be discussed how the relevance of endpoints for patients can be incorporated more strongly in EBA in the future. In this respect to the set-up of treatment guidelines, both clinical experts appointed by clinical professional societies and affected patients should be consulted in order to achieve a large degree of acceptance in society.

**Table 2:** Levels of patient-relevant benefit, as per German Social Code

	
**1**	**Major** (“erheblich”)–*Lasting major improvement*
	–*Especially recovery from the disease, a significant extension in the duration of survival, long-term freedom from severe symptoms or extensive avoidance of serious side effects*
**2**	**Important** (“beträchtlich”)–*Significant improvement*
	–*Especially a reduction of serious symptoms, a moderate extension of the duration of life, an alleviation of the disease that is noticeable for the patients, a relevant avoidance of serious side effects, or a significant avoidance of other side effects*
**3**	**Slight** (“gering”)–*Moderate and not just minor improvement*
	–*Especially a reduction of non-serious symptoms of the disease or a relevantavoidance of side effects*
**4**	Existing; but not quantifiable
	–*Because the scientific data basis does not allow such quantification*
**5**	None
**6**	Smaller *than the benefit of the appropriate comparator*

Survival of patients without their disease getting worse (PFS) is suitable as an endpoint in many oncological therapies, not just for survival but–apart from other target variables such as tumor response or the duration of tumor response–also as a morbidity-related, separate endpoint. A significant improvement of PFS with acceptable side effects demonstrates a clinically relevant, valid PRB in terms of morbidity (defined as the problems and complications of a disease) especially in maintenance treatment and palliative situations. In RCC, for example, in the context of disease progression, not only new metastases can occur, but also already existing metastases can progress [19,20]. Last but not least, the (perhaps not resectable) primary tumor can deteriorate further. This can be associated with symptoms and may require procedures that directly burdens the individual patient [21,22]. PFS per se is of immediate, clinically and patient-relevant value in these patients [23]. Furthermore, the patient can be bothered by fear of progression. In contrast to irrational fears, fear of progression is real and is apperceived by the individual patients: Herschbach and colleagues have demonstrated that in about one third of patients are distressed or highly distressed due to fear of progression [24]. Finally, the importance of progression for the patients has also been demonstrated by Cella and Colleagues who found a relatively stable course of HRQoL during progression-free treatment period, and a dramatic decline in case of progression [25].

### Practical relevance: the case of imatinib and its implications

The practical relevance of such an approach is shown by the case of imatinib for treatment of CML. Today, imatinib is indisputably considered a breakthrough innovation in CML therapy. It has to be noted imatinib received EMA MA based on PFS data.

In the pivotal trial for imatinib, [26] a significant benefit for imatinib compared to the standard therapy (interferon-alpha + cytarabine) was proven for an overall population of 1,106 patients after 19 months in terms of PFS. In the case of non-response to treatment with the standard therapy, a cross-over to imatinib was possible.

In retrospect, the cross-over in the International Randomized Study of Interferon and STI571 (IRIS) study also showed the innovative potential of imatinib: [26]. One trend for an OS benefit (post-hoc, p = 0.075) could only be demonstrated for imatinib vs. standard therapy through long-term evaluations after six years of follow-up by Hochhaus and Co-workers [27]. For the German Society for Hematology and Oncology (DGHO), Ehninger and Wörmann further demonstrated that for this study population during a six year time period, about 332 additional patients would have died in the comparative arm alone, had they been denied a cross-over to imatinib [28].

For ethical reasons alone, OS must not be the sole valid endpoint for EBA of anticancer medicines. In the case of the breakthrough innovation imatinib, if OS would have been accepted as the only PRE, the drug may have only been reimbursed after six years of availability in the German market. For NSCLC, patients are often treated with an individualized, marker-based treatment approach with medicines that received MA via a primary PFS endpoint. In this indication, an increase of OS rates has also been reported, confirming the positive trend PFS had previously shown [29].

Apart from PFS, the parameters of (duration of) tumor response to therapy should be taken into account during the evaluation of patient relevance in case regulatory authorities have accepted these endpoints during evaluation of benefit-risk ratio.

The disappearance, shrinkage or stabilization of a tumor is also associated with a direct benefit for the patient in many indications (such as lung cancer), e.g. based on (i) Reduction or at least stabilization of tumor-related symptoms (e.g. shortness of breath, congestion/compression due to the tumor); [30] (ii) Delay of subsequent treatment with additional side effects; [31] (iii) Stabilization of the disease, as experienced positively by the patient (“treatment is effective”) [32] and (iv) Possibility of surgical intervention or improvement of the result of surgery (e.g. neo-adjuvant treatment concept for invasive mamma carcinoma) [33]. In addition, patient surveys for e.g. NSCLC showed that in the progression of an advanced disease the relevance of tumor and symptom control is becoming more important and that therapy for improving tumor symptoms is desired, even if an extension of survival can no longer be achieved [34].

Other PRE for morbidity include time to treatment failure or cytogenetic complete remission rate. It must also be considered that progression of a tumor disease is typically associated with a deterioration of the general condition and/or the disease-related symptoms, which requires a change in therapy. A large number of publications are concerned with the aspect of the direct influence of non-mortality-related parameters on the subjective, patient-experienced disease situation [35-40].

IQWiG recently also followed the argument that complete remission is also patient-relevant as an ultimate target-variable in hematologic malignancies if it coincides with less morbidity or improved HRQoL [41]. The authors believe that a broad perspective must be applied to the endpoints of oncological studies and the chosen endpoints from the clinical trial should be recognized in the EBA in individual cases. PFS in terms of a morbidity parameter and other disease-related endpoints also constitute valid PREs for EBA and must be used appropriately and comprehensively. Otherwise, EBA according to the state of science and in compliance with the Volume V of the German Social Code would be impossible for many oncological pharmaceuticals.

It should be mentioned, that the value of study-endpoints for the affected patients can be attained through evaluation of patient preferences. For the malignant disease of plasmocytoma/multiple myeloma, Mühlbacher and Nübling were able to demonstrate that, apart from the effect of a treatment, patients in conjoint-analyses rate other endpoints as equally important for their subjective disease experience [42]. These preferred factors, from a total of 16 polled endpoints, included primarily a long-lasting effect of the therapy, disease-free duration of life and therapy breaks. Noteably, these three latter endpoints are weighted more strongly by the patients than an extension of OS alone. In order to meet this explicit patient wish, it is indispensable that PFS as a morbidity parameter must be viewed as imminently useful and valuable to the affected patient. Guidances or recommendations set up by relevant scientific societies need to be taken into account in the benefit assessment.

### Summary

**Figure 2:**
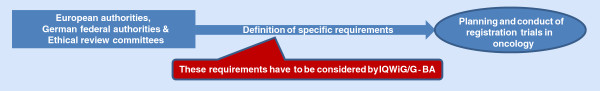
Task force position flow chart.

In summary, for proving an additional PRB in oncology, morbidity endpoints must be considered in addition to mortality and HRQoL in order to adequately address the complexity of the situation of the individual patient and his or her treating physicians (see Figure 2).

In principle, these endpoints must be viewed on a par according to the German Social Code. A gradation of the PRB, depending on whether or not a gain in OS was proven, is logically untenable, since the possibility of the proof itself–as described–depends in many cases on the tumor entity, the tumor stage and the individual situation of the patient and not just on the pharmaceutical alone. In this context, it must be emphasized once more that a definition of the term “patient relevance” and a final weighting of endpoints is still pending. As a consequence, it must also be discussed how the relevance of endpoints for the patient can enter EBA more strongly in the future. Apart from clinical experts, the affected patients in particular should also be consulted in this respect. In accordance with the development of treatment guidelines, both, clinical experts appointed by clinical professional societies, and affected patients should be consulted in order to achieve a large degree of acceptance in society.

A close review of the individual case is necessary in the operationalization of the PRB. While the G-BA’s wish for a standardized assessment is understandable, the example of imatinib for the treatment of CML [26-28] illustrates nonetheless that the model currently under discussion would not have properly assessed even a recognized/established breakthrough innovation as such.

## Conclusions

Depending on tumor entity and tumor stage, the endpoints for a PRB are not necessarily identical. In this respect, all parties involved match in their statements.

EBA must not result in a loss of perception of the disease-and stage-specific therapy needs based on methodological simplification. Apart from endpoints assessing survival time such as OS and PFS, endpoints regarding morbidity in terms of symptoms and complications of a disease as well as HRQoL also represent endpoints for PRB in pivotal studies, are recognized by regulatory agencies and must also be accepted in principle for EBA. Apart from that, clinical-scientific findings and guidelines as well as patient needs must be considered appropriately and comprehensively.

The basic benefit of a new cancer therapy is proven during the MA procedure and represents the basis of the MA decision [2]. Therefore, as part of the EBA, the aspects of clinical research and international regulatory MA experience must be incorporated in order to avoid a separate German way. As a result, a broad and comprehensive discussion with scientific experts as part of the EBA is needed. This should help to avoid the emergence of demands or the representation of opinions that miss the mark regarding the needs of today’s society and specifically the reality of medical research. Not taken into account in this respect are ethical aspects of clinical research and the needs of the patients.

If this path is not taken, new developments and therapeutic approaches in oncology in Germany would be faced with unsubstantiated obstacles regarding the early benefit assessment, which cannot be in the society’s interest.

## Competing interest

Employment at the research period of the project: ID, C-MD, and TR – German Association of Research-based Pharmaceutical Companies (vfa); JD – GlaxoSmithKline GmbH & Co. KG, Munich, Germany; MD – Boehringer Ingelheim Pharma GmbH & Co. KG, Ingelheim, Germany; DK – Pfizer Pharma GmbH, Berlin, Germany; PK – MSD SHARP & DOHME GMBH, Haar, Germany; FL – Lilly Deutschland GmbH, Bad Homburg, Germany; JR – Roche Pharma AG, Grenzach-Wyhlen, Germany; DW – Janssen-Cilag GmbH, Neuss, Germany.

## Authors’ contributions

PK, JR and TR made substantial contributions to conception and design of the study. ID, JD, MD, DK, PK, and DW made substantial contributions to creation of text blocks and tables. PK and TR were involved in drafting the manuscript. ID, JD, C-MD, MD, DK, PK, FL, JR, TR, and DW made substantial contributions to creation of the final manuscript. PK, TR and DW collected stakeholder input. C-MD, FL and DW revised the final draft of the manuscript critically for important intellectual content. All authors gave final approval of the version to be published.

## Supplementary Material

Additional file 1: Table S1 Outcomes of German HTA early benefit assessments in Oncology, 2011-2013 (all oncological appraisals with at least oral G-BA hearing before Apr-1st, 2013 included). Click here for file
