# Supplementary material for: Patient relevant endpoints in oncology: current issues in the context of early benefit assessment in Germany
Source: Health Econ Rev. 2014 Jan 24;4:2. doi: 10.1186/2191-1991-4-2 (PMC3901346; doi:10.1186/2191-1991-4-2)
Supplement: Additional file 1: Table S1 — Outcomes of German HTA early benefit assessments in Oncology, 2011-2013 (all oncological appraisals with at least oral G-BA hearing before Apr-1st, 2013 included). [file 2191-1991-4-2-S1.pdf]

Table 2: Outcomes of German HTA Early Benefit Assessments in Oncology, 2011-2013 (all oncological appraisals with at least oral G-BA hearing before Apr-1st, 2013 included).

				IQWiG[o]								G-BA[p]			
No.	Generic name (Brand name, pharmaceutic entrepreneur)	EU Market authorizations (EMA)	Adequate comparator as defined by G-BA	Subgroups according to IQWiG (Patient groups)	Mortality	Morbidity	Quality of Life	Side effects	Cure	Patient-relevant benefit[2]	Extent of patient-relevant benefit[1]	(i) Status of appraisal and (ii) time limitation	Endpoints contributing to final extent of overall added benefit	Patient-relevant benefit according to G-BA[2,1]	Relevant subgroups according to G-BA
1	Abiraterone-acetate (Zytiga®, Janssen)	Metastatic castration resistant prostate cancer (MCRPC), progressed on or after a docetaxel-based chemotherapy regimen[a]	Palliative treatment with dexamethasone, or (methyl) predniso(lo)ne, or BSC, respectively	1. BSC population[l]	OS - B	Time to first skeletal injury - B<br>Time to pain progression - B	F[n]	AE, AE CTCAE Grade 3/4, sAE, AE leading to treatment discontinuation or death - D	Not assessed	Indication	Important	(i) Closed<br>(ii) None	OS- L<br>Time to first skeletal injury - N<br>Time to pain progression - N<br>Safety - N	Indication<br>Important (2)	1. MCRPC progressing during or after docetaxel therapy, not qualifying for docetaxel retreatment
			Docetaxel + predniso(lo)ne	2. Docetaxel-re-treatment polulation[m]	F	F	F	F	Not assessed	None	None			None (5)	2. MCRPC progressing during or after docetaxel therapy but that still qualify for docetaxel retreatment
2	Cabazitaxel (Jevtana®, Sanofi)	In combination with predniso(lo)ne for the treatment of hormone refractory metastatic prostate cancer (MPC) previously treated with a docetaxel-containing regimen[b]	Mitoxantrone + predniso(lo)ne	1. BSC population[l]	OS - B (pts aged-65+)<br>OS - C (pts aged-64 or younger)	PRO/Pain - D	F	AE, AE CTCAE Grade 3/4, sAE, AE leading to treatment discontinuation or death - E	Not assessed	1. Indication (pts aged-65+)<br>2. Hint (pts aged-64 or younger)	1. Important (pts aged-65+)<br>2. Not quantifiable (pts aged-64 or younger)	(i) Closed<br>(ii) None	OS- L<br>Safety - D	Indication<br>Slight (3)	1. Hormone-refractory MPC suffering progressing during or after docetaxel-containing chemotherapy not qualifying for docetaxel retreatment
			Docetaxel + predniso(lo)ne	2. Docetaxel-Re-treatment polulation[m]	F[n]	F	F	F	Not assessed	None<br>(no data available)	None		None	None (5)	2. Hormone-refractory MPC progressing during or after docetaxel-containing chemotherapy qualifying for docetaxel retreatment
3	Eribulin (Halaven®, Eisai)	Locally advanced or metastatic breast cancer (MBC) after at least two chemotherapeutic regimens for advanced disease ... Prior therapy should have included an anthracycline and a taxane unless pts were not suitable for these treatments.[c]	Monotherapy with capecitabine, 5-Fluorouracil, or vinorelbine, respectively	1. MBC no longer eligible for therapy with taxanes or anthracyclines	OS - C	F	F	AE, AE CTCAE Grade 3/4 - E<br>sAE, AE leading to treatment discontinuation or death - D	Not assessed	None	PRB with regard to OS not quantifiable vs major harm due to side effects (which cannot be excluded)	(i) Closed<br>(ii) 2 Years (Apr-19th, 2014)	OS - L<br>Safety - D	Hint<br>Slight (3)	1. MBC no longer eligible for therapy with taxanes or anthracyclines
			Retreatment with anthracycline- or taxane-containing therapy	2. MBC eligible for retreatment with taxanes or anthracyclines	OS - D	F	F	AE, AE CTCAE Grade 3/4 - E<br>sAE, AE leading to treatment discontinuation or death - D	Not assessed	None	None		OS - N<br>Safety - D	Hint<br>Smaller (6)	2. MBC eligible for retreatment with taxanes or anthracyclines
4	Ipilimumab (Yervoy®, BMS)	Advanced (unresectable or metastatic) melanoma in adults who have received prior therapy.[d]	BSC	Advanced (unresectable or metastatic) melanoma after prior therapy	OS - A	F	PRO/HRQoL - D	AE, AE CTCAE Grade 3/4, sAE - No PRB (numerical differences only)	Not assessed	Indication	Important	(i) Closed<br>(ii) 5 Years (Aug-2nd, 2017)	OS - L<br>PRO/HRQoL - N<br>Safety - N	Indication<br>Important (2)	Advanced (unresectable or metastatic) melanoma after prior therapy
5	Vemurafenib (Zelboraf®, Roche)	BRAF V600 mutation-positive unresectable or metastatic melanoma in adults[e]	Dacarbazine (DTIC)	BRAF V600 mutation-positive unresectable or metastatic melanoma	OS - A	PRO/Pain - D	PRO/HRQoL - D	AE - D	Not assessed	Indication	Important	(i) Closed<br>(ii) 1 Year (Sep-6th, 2013)	OS - L<br>PRO/Pain - N<br>PRO/HRQoL - N<br>Safety - N	Indication<br>Important (2)	BRAF-V600 mutation-positive, unresectable or metastatic melanoma
6	Vandetanib (Caprelsa®, AstraZeneca)	Unresectable locally advanced or metastatic aggressive and symptomatic medullary thyroid cancer (MTC).[f]	BSC	-	F[n]	F[n]	F[n]	F[n]	Not assessed	None<br>(Dossier incomplete)	None	(i) Closed<br>(ii) None	None	None (5)	Unresectable, locally advanced or metastatic aggressive and symptomatic MTC
7	Tegafur/Gimeracil/Oteracil (Teysuno®, Nordic Pharma)	Advanced gastric cancer, in combination with cisplatin[g]	Cisplatin combined with 5-fluorouracil or capecitabine	-	n/a[3]	n/a[3]	n/a[3]	n/a[3]	n/a[3]	n/a[3]	n/a[3]	(i) Closed<br>(ii) None	None	None (5)	Advanced gastric cancer (in combination with cisplatin)
8	Axitinib (Inlyta®, Pfizer)	Advanced renal cell carcinoma (RCC) after failure of prior treatment with sunitinib or a cytokine.[h]	Sorafenib	1. Adult patients with advanced renal cell carcinoma (RCC) after failure of prior treatment with a cytokine	OS - D	PRO/Symptoms - D	PRO/HRQoL - D	AE, AE CTCAE Grade 3/4, sAE, AE leading to treatment discontinuation or death - D<br>Alopecia, Rash, Hand-foot-syndrome - B	Not assessed	Hint	Important	(i) Closed<br>(ii) 4 Years (Mar-21, 2017)	OS - N<br>PRO/Symptoms - N<br>PRO/HRQoL - N<br>Safety - L	Indication<br>Minor (3)	1. RCC after failure of prior treatment with a cytokine
			Everolimus	2. Adult patients with advanced renal cell carcinoma (RCC) after failure of prior treatment with sunitinib	OS - D	PRO/Symptoms - D	PRO/HRQoL - D	AE - D<br>AE CTCAE Grade 3/4, sAE, AE leading to treatment discontinuation or death - F	Not assessed	None	None		None	None (5)	2. RCC after failure of prior treatment with sunitinib
9	Decitabine (Dacogen®, Janssen)	Pts aged 65 years and above with newly diagnosed de novo or secondary acute myeloid leukemia (AML), according to the WHO classification, who are not candidates for standard induction chemotherapy.[j]	As defined by pharmaceutical entrepreneur (Orphan drug)	-	n/a[34]	n/a[34]	n/a[34]	n/a[34]	n/a[34]	n/a[34]	n/a[34]	(i) Closed<br>(ii) None	OS - L<br>Safety - N	Minor (3)	Newly diagnosed de novo or secondary AML pts aged 65 years and above
10	Crizotinib (Xalkori®, Pfizer)	Previously treated anaplastic lymphoma kinase (ALK)-positive advanced non-small cell lung cancer (NSCLC).[k]	Docetaxel / Pemetrexed	1. Pre-treated ALK-positive, advanced NSCLC where a chemotherapy is indicated (ECOG 0, 1 and possibly 2)	OS - D	F[n]	PRO/HRQoL - C	AE, AE CTCAE Grade 3/4, AE leading to treatment discontinuation - D<br>Visual disturbances, gastrointestinal events, sAE - E	None	None	None	(i) Closed<br>(ii) 2 years (May-2nd, 2015)	OS - N<br>PRO/Symptoms - L<br>PRO/HRQoL - L<br>Safety - N	Hint<br>Important (2)	1. Pre-treated ALK-positive, advanced NSCLC where a chemotherapy is indicated
			BSC	2. Pre-treated ALK-positive, advanced NSCLC where a chemotherapy is not indicated (ECOG 4, 3 and possibly 2)	F[n]	F[n]	F[n]	F[n]	None	None<br>(no relevant Data)	None		None	None (5)	2. Pre-treated ALK-positive, advanced NSCLC where a chemotherapy is not indicated

All Appraisals with at least oral G-BA hearing before Apr-1st, 2013.

AE=Adverse event. BSC=Best supportive care. CTCAE=Common terminology criteria for adverse events. ECOG=Eastern Cooperative Oncology Group Performance Status. EMA=European Medical Agency. G-BA=German Joint Federal Committee (Gemeinsamer Bundesausschuss). HRQoL=Health related quality of life. IQWiG=German Institut für Qualität und Wirtschaftlichkeit im Gesundheitswesen. OS=Overall survival. PRB=Patient relevant benefit as defined by German Social Code (Book V). PRO=Patient reported outcomes. PTs=Patients

A=Proof of PRB. B=Indication of PRB. C=Hint of PRB. D=No PRB (numerical differences only). E=Harm. F=No data. L=Affirmative for level of benefit. N=Neutral. D=Leads to devaluation

[1] Additional medical benefit ("Zusatznutzen") relative to comparator assigned by G-BA: 1 - Major ("erheblich"), Lasting major improvement. 2 - Important ("beträchtlich"), Significant improvement; 3 - Slight ("gering"), moderate and not just minor improvement; 4 - Not quantifiable, exisitng, but not quantifiable; 5 - None; 6 - Smaller than the benefit of the appropriate comparator.

[2] Categories: Proof ("Beweis"), highest possible; Indication ("Hinweis"); Hint ("Anhaltspunkt") - see IQWiG Methods (https://www.iqwig.de/en/methods/methods_papers/general_methods.3020.html, accessed 04Apr2013)

[3] Assessment performed by G-BA

[4] Orphan drug

[a] http://www.ema.europa.eu/ema/index.jsp?curl=pages/medicines/human/medicines/002321/human_med_001499.jsp&mid=WC0b01ac058001d124, accessed 04Apr2013

[b] http://www.ema.europa.eu/docs/en_GB/document_library/EPAR_-_Product_Information/human/002018/WC500104764.pdf, accessed 04Apr2013

[c] http://www.ema.europa.eu/docs/en_GB/document_library/EPAR_-_Product_Information/human/002084/WC500105112.pdf, accessed 04Apr2013

[d] http://www.ema.europa.eu/docs/en_GB/document_library/EPAR_-_Product_Information/human/002213/WC500109299.pdf, accessed 04Apr2013

[e] http://www.ema.europa.eu/docs/en_GB/document_library/EPAR_-_Product_Information/human/002409/WC500124317.pdf, accessed 04Apr2013

[f] http://www.ema.europa.eu/docs/en_GB/document_library/EPAR_-_Product_Information/human/002315/WC500123555.pdf, accessed 04Apr2013

[g] http://www.ema.europa.eu/docs/en_GB/document_library/EPAR_-_Product_Information/human/001242/WC500104415.pdf, accessed 04Apr2013

[h] http://www.ema.europa.eu/docs/en_GB/document_library/EPAR_-_Product_Information/human/002406/WC500132188.pdf, accessed 04Apr2013

[j] http://www.ema.europa.eu/docs/en_GB/document_library/EPAR_-_Product_Information/human/002221/WC500133569.pdf, accessed 04Apr2013

[k] http://www.ema.europa.eu/docs/en_GB/document_library/EPAR_-_Product_Information/human/002489/WC500134759.pdf, accessed 04Apr2013

[l] prostate cancer patients that progressed during or after docetaxel therapy and that do not qualify for retreatment with docetaxel

[m] prostate cancer patients that progressed during or after docetaxel therapy but that still qualify for retreatment with docetaxel

[n] data classified as not usable

[o] https://www.iqwig.de/de/projekte_ergebnisse/projekte.1057.html, accessed 04Apr2013

[p] http://www.g-ba.de/informationen/nutzenbewertung/, accessed 12Dec2013
